# Fractional Solitons in Excitonic Josephson Junctions

**DOI:** 10.1038/srep15796

**Published:** 2015-10-29

**Authors:** Ya-Fen Hsu, Jung-Jung Su

**Affiliations:** 1 Department of Electrophysics, National Chiao Tung University, Hsinchu 300, Taiwan

## Abstract

The Josephson effect is especially appealing to physicists because it reveals macroscopically the quantum order and phase. In excitonic bilayers the effect is even subtler due to the counterflow of supercurrent as well as the tunneling between layers (interlayer tunneling). Here we study, in a quantum Hall bilayer, the excitonic Josephson junction: a conjunct of two exciton condensates with a relative phase *ϕ*_0_ applied. The system is mapped into a pseudospin ferromagnet then described numerically by the Landau-Lifshitz-Gilbert equation. In the presence of interlayer tunneling, we identify a family of *fractional* sine-Gordon solitons which resemble the static fractional Josephson vortices in the extended superconducting Josephson junctions. Each fractional soliton carries a topological charge *Q* that is not necessarily a half/full integer but can vary *continuously*. The calculated current-phase relation (CPR) shows that solitons with *Q* = *ϕ*_0_/2*π* is the lowest energy state starting from zero *ϕ*_0_ – until *ϕ*_0_ > *π* – then the alternative group of solitons with *Q* = *ϕ*_0_/2*π* − 1 takes place and switches the polarity of CPR.

Excitons, the electron-hole pairs bound by Coulomb interaction, can reach spontaneous phase coherence and form excitonic supercurrent^1^,^2^,^3^,^4^,^5^,^6^,^7^,^8^,^9^,^10^ when properly induced^11^. They are in close analogy to the Cooper pairs in s-wave superconductors in that both can be described by the SU(2) BCS-type theory^12^. The overall charge neutrality of excitons, however, requires that the electron- and hole-components to be spatially separated for the electrical current detection^1^; the separation should be sufficiently small to maintain the excitonic coherence. Thank to the advance of semiconductor manufacturing technology, excitonic superfluid in such geometry is readily realized in Quantum Hall bilayers (QHBs)^9^,^10^,^13^. Unique effects for excitons in bilayer include the interlayer tunneling anomaly^14^,^15^,^16^,^17^,^18^,^19^,^20^,^21^ and the current counterflow^17^,^22^,^23^,^24^,^25^,^26^,^27^. Both offer exotic twists to the already fascinating supercurrent phenomena – among all the *Josephson effect*.

First proposed and demonstrated in superconductor^28^, Josephson effect is regarded as an unambiguous test to superfluidity or superconductivity. The dc Josephson effect, in particular, describes the *zero-bias* supercurrent occurring in a *Josephson junction* – a device consisting of two coupled condensates with a relative phase applied. The Josephson effect is usually characterized by its current-phase relation (CPR)^28^,^29^. Although best known in the standard sinusoidal form, the CPR can go beyond sinusoids to genuinely reflect the junction geometry and the composite material’s properties^29^. In the context of exciton condensation, the attention has been on a seemingly similar but practically different effect, the *Josephson-like effect*^14^,^15^,^16^,^17^,^18^,^19^,^20^,^21^,^30^,^31^,^32^,^33^,^34^,^35^,^36^,^37^,^38^. The few pioneering works^39^,^40^,^41^,^42^ that are actually on the Josephson effect (dc), however, have yet included the interlayer tunneling in dynamics; the sinusoidal Josephson relation is thus applied directly. Here we actively include the interlayer tunneling in the equation of motion to obtain the appropriate CPR for the excitonic Josephson junction. It turns out that the obtained CPRs actually go beyond the standard sinusoidal form. Moreover, the *fractional solitons* emerge in static. Similar solitons with exactly half quanta have been realized in only few specially designed superconducting systems^43^,^44^,^45^,^46^,^47^. Such half-integer solitons generated in a controllable fashion can be strong candidates for quantum qubits^44^. The fractional solitons we discuss here are even more profound: it embraces *continuously varying fractions* that are not limited to half or full integers^46^,^47^. Because of the continuously varying nature, abrupt occurrence of solitons with increasing relative phase *ϕ*_0_ is not observed – the solitons appear progressively starting from infinitesimal *ϕ*_0_.

## Methods

### System setup

**Figure 1:**
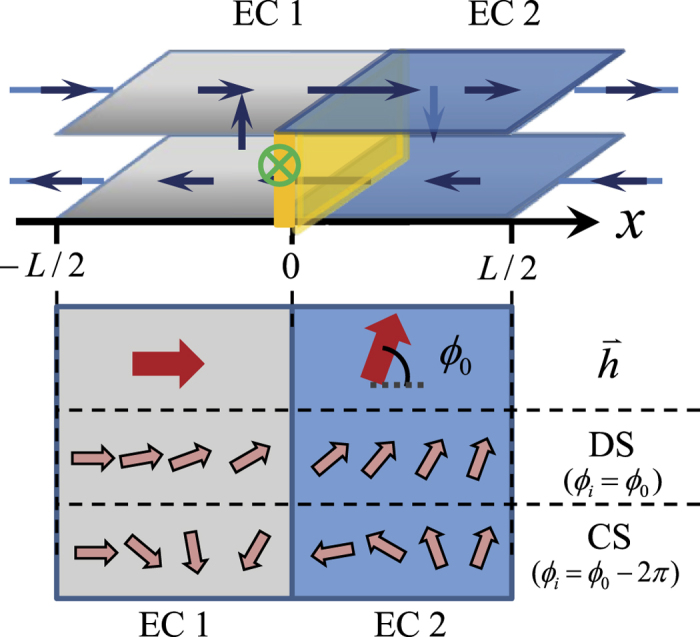
Schematic illustration of the excitonic Josephson effect. In the counterflow geometry (upper half), a relative phase *ϕ*_0_ is generated between the two excitonic condensates, EC1 and EC2. The navy arrows indicate the current flows. Note that currents are allowed to flow between layers while the net current should conserve. Lower half of Fig. 1 demonstrates the pseudospin picture of the excitonic Josephson effect. The relative phase *ϕ*_0_ corresponds to an angle difference in two pseudospin Zeeman fields (red arrows). The Zeeman fields attempt to work against the pseudospin stiffness to align the local pseudospins (pink arrows) with themselves. There are two configurations, a direct solution (DS) and a complementary solution (CS) that correspond to incline angles equal *ϕ*_0_ and *ϕ*_0_ − 2*π*, respectively.

The excitonic Josephson junction we consider is illustrated in Fig. 1 (upper half). In an excitonic bilayer, the two constituent layers are separately connected to allow counterflow; blue arrows in the figure show antiparallel current flow. Interlayer tunneling is represented by electron-current flow between the two layers. This tunneling should conserve the sum of current in the two layers. A constant relative phase *ϕ*_0_ is then applied between the two excitonic condensates, EC1 and EC2. By applying *interlayer* voltage pulse on EC2, we can prepare the junction at a relative phase *ϕ*_0_^39^,^40^,^48^. Such a designated *ϕ*_0_ is reached by controlling the magnitude or the duration of the applying voltage pulse. In the absence of *lateral* electric field is zero, the edge current would not play a role in the present discussion. We detail in the following sections how the system is treated by solving the Landau-Lifshitz-Gilbert (LLG) equation^49^ for pseudospins^50^. This approach is especially powerful in mapping out the pseudospin distribution and thus the phase profile.

### Pseudospin model and the equation of motion

We begin with the excitonic wave function in the quantum Hall bilayer^6^


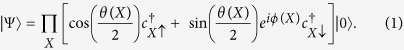


The ↑ (↓) denotes the *which layer* degree of freedom and *X* the guiding center quantum number of the lowest Landau level. Vacuum state 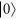 indicates no electron in either layer. When representing 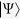 as a vector in the Bloch sphere, *θ*(*X*) and *ϕ*(*X*) become the associated polar and azimuthal angles. Such a vector is hereafter referred to as the *psuedospin* 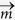 (classical) – the exciton system is ultimately mapped to a pseudospin ferromagnet. In the limit of smooth textures, the energy functional can be expressed by pseusospin 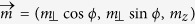:


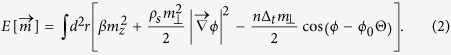


The first term is the capacitive energy deduced from the Hartree and intralayer exchange interactions. The positive anisotropic parameter *β* means an energy cost for the pseudospin out-of-plane excursion. This term is neglected from now on since we focus on the condensation regime in this report. The second term is essentially the kinetic energy of excitonic condensates which arises from the interlayer exchange. In the pseudospin language, the positive *ρ*_*s*_ tends to align neighboring pseudospins in parallel and serves as the pseudospin stiffness.

The third term, namely the interlayer tunneling, is the key to our work. Here *n* and Δ_*t*_ are the pseudospin density and the interlayer tunneling strength respectively. Aside from the ordinaries, this term contains an extra *ϕ*_0_Θ(*x*) [Θ(*x*) is the standard Heaviside function]. Note that in arriving at this expression we have implicitly taken the absolute phase of EC1 to be 0, without losing generality. The relative phase *ϕ*_0_ is originated from the Aharnov-Bohm phase when the electrons circle around a loop with effective magnetic flux. This form of interlayer tunneling can be interpreted as the pseudospin Zeeman term: 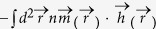, with the Zeeman field 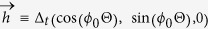, which attempts to align the local pseudospins 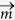 with itself.

### Equation of motion

We derive the Landau-Lifshitz-Gilbert (LLG) equation for pseudospins by 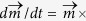 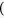
*nh–*/2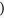 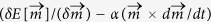
49, where 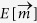 is defined in Eq. (2). Pseudospin in general has two degrees of freedom; we pick *ϕ* and *m*_⊥_ to describe. Two coupled equations are then solved numerically by our LLG solver, detailed in ref. 50.

In parallel, we minimize the energy functional in Eq. (2) with respect to *ϕ* to acquire the static properties. An equation that resembles the sine-Gordon equation is obtained:


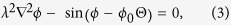


where 
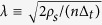
 is the Josephson length that sets the spatial scale of topology. The phase *ϕ* is apparently a function of the relative phase *ϕ*_0_. However for a given *ϕ*_0_, the solution is not unique: replacing *ϕ*_0_ by *ϕ*_0_ + 2*Nπ* in a known solution gives rise to a set of distinct valid solutions (not all are stable). To describe the situation, we denote the phase profile *ϕ* as a function of the *incline angle* 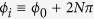 instead of *ϕ*_0_ itself. Among all, the *direct solution* (DS) and *complementary solution* (CS) are energetically most relevant. Here we illustrate the two in the pseudospin picture: a given relative phase *ϕ*_0_ determines the direction of the pseudospin Zeeman field 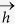 as depicted in Fig. 1 (the absolute phase of EC1 is set to zero). The same Zeeman field is however associated with various *incline angles* 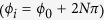. Taking *ϕ*_*i*_ = *ϕ*_0_, the local pseudospins then wind *counter-clockwise* (for positive *ϕ*_0_), seeking to be commensurate with both the Zeeman fields and yields the “direct solution” (plotted in row marked as “DS”). Taking *ϕ*_*i*_ = *ϕ*_0_ − 2*π*, on the other hand, the local pseudospins then wind *clockwise* to yield the “complementary solution” (CS). As shown in Fig. 1, the two solutions exhibit opposite polarities and even more, are generally *not* mirror symmetric.

## Results

### Phase profiles and fractional sine-Gordon Solitons

**Figure 2:**
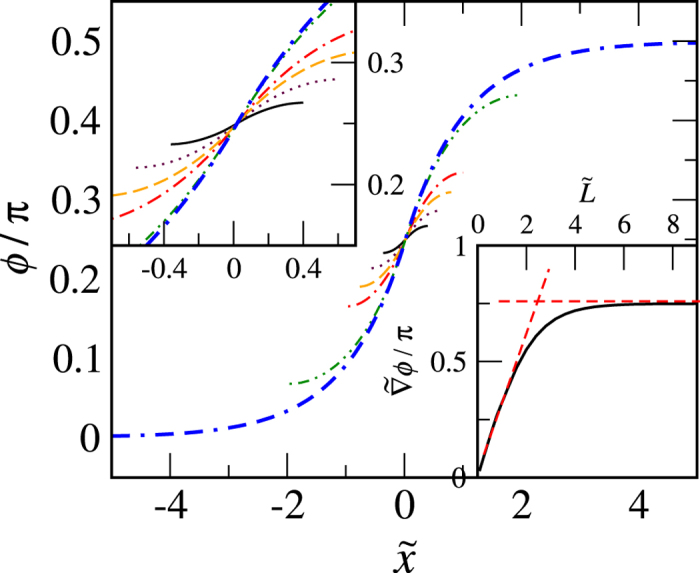
Typical phase profiles of *ϕ*_*i*_ = *π*/2 for different system sizes including 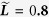 (black solid line), 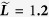 (purple dotted line), 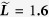 (orange dash line), 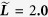 (red dash-dotted line), 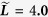 (green dash-dotted-dotted line), 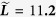
 **(blue dash-dash-dotted line).** The upper-left inset is the zoom-in of the small 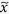 region that shows deviations of 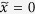 phase slope for short systems. The lower-right inset is the phase slope vs. the system size 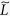. For short systems 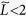, the slopes are linear with 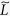 while for long junctions 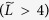 they are practically a constant. The two red dash lines are guide to eyes.

The phase profile 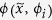 is a function of both the position 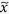 and the incline angle *ϕ*_*i*_ as mentioned. We first show the static phase profiles, which is obtained by solving LLG equation with *ϕ*_*i*_ = *π*/2 for various system sizes (main body of Fig. 2). A widely accepted set of parameters for the total filling number *v*_*T*_ = 1 QHB are used in this and in all later calculations (parameters expressed as dimensionless quantities): 
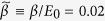
, 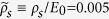 and 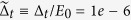, with the energy unit 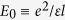 (the range of these parameters are nicely summarized in ref. 38). All lengths hereafter are also dimensionless quantities taken with respect to the Josephson length *λ*.

The phase profiles in the short and the long junctions are visibly different although both exhibit odd symmetry about 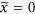 and 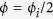 (Fig. 2). The shape is practically linear in short junctions while saturates at both ends in long junctions. The zoom-in in the vicinity of 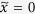 (upper-left inset) further shows that the profiles from the two limits deviate already at small 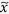: the shorter junctions tend to possess gentler slopes. The slope vs. system size plot (lower-right inset) demonstrates that tendency and shows a smooth crossover at 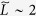. The behavior evolves from *linear* to *constant* with ascending 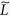. Below we analyze the detailed profiles in the two limits.

In the short junctions, 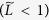, the phase profiles do not extend fully from 0 to *ϕ*_0_ and are reasonably described by a slanting line 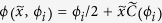 [here 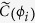 is the slope]. By minimizing the system’s total energy with respect to 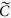, we obtain the profile


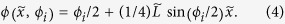


The short-junction assumption 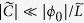 has been employed in reaching this expression. Note especially that the corresponding phase slope 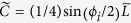 is linearly proportional to the junction length, recovering the numerical result. The energy of the entire junction associated with the slanting phase profile is


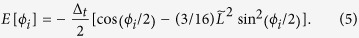


We can deduce, from the above, difference between the CS and the DS energy is Δ_*t*_cos(*ϕ*_0_/2). The two come to degenerate when *ϕ*_*i*_ equals multiples of *π*.

**Figure 3:**
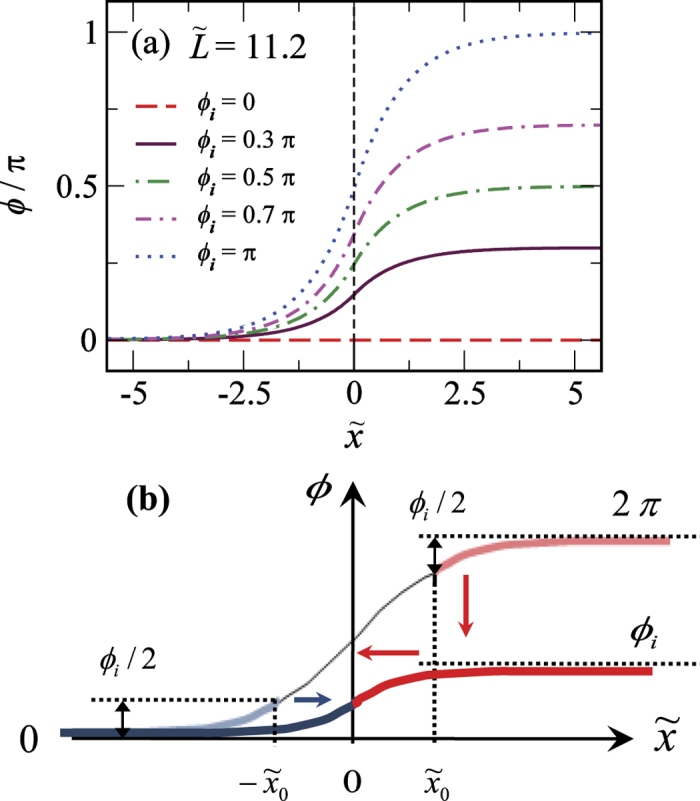
(**a**) Phase profile for various values of *ϕ*_*i*_ of a fixed system length 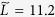. Fractional solitons are developed in all profiles. (**b**) Cartoon for constructing fractional soliton. Two designated sections of a full sine-Gordon soliton, one from left (blue) and the other from right (red), are shifted to joint at 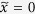 and form a *fractional soliton*.

In the long junctions 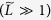, the phase profiles show *kinks* that span between 0 and *ϕ*_*i*_ [Fig. 3(a)]. Those are *fractional sine-Gordon solitons* that can be constructed from the ordinary sine-Gordon solitons [Fig. 3(b)]: The ultimate guideline are the boundary conditions, 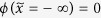 and 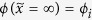. With the ordinary sine-Gordon soliton at hand, we first shift its tail left to 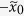 (light blue curve) rightward by 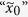. The tail right to 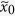 (light red curve) is then shifted first downward by “2*π* − *ϕ*_0_” then leftward by 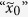. No shrinking or stretching should be involved in the entire process. The landmark 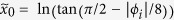 is given such that 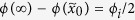. The profile for the fractional soliton is then literally:


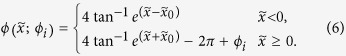


A definite topological charge 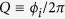 can be assigned to characterize the solitons; this charge is closely related to the *winding number* that describes a vortex. It is interesting to note that similar geometric objects (fractional Josephson vortices) have been predicted also in an extended 0 − *κ* superconducting Josephson junction^46^,^47^.

The energy associated with the fractional soliton in a long junction is then given by Eqs. (2) and (6):


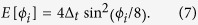


With *ϕ*_*i*_ = 2*π*, the energy *E* = 2Δ_*t*_ is that of an ordinary soliton. This is a direct consequence of the equipartition theorem if interpreting Δ_*t*_ as the potential energy. Finally we remark that although Eq. (7) might appear as a nonmonotonic function of *ϕ*_*i*_, it actually is the opposite – there is no soliton solution for 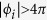. The construction above is impossible for 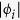 exceeding 4*π* because a meaningful 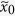 does not exist.

### Current-phase relation

**Figure 4:**
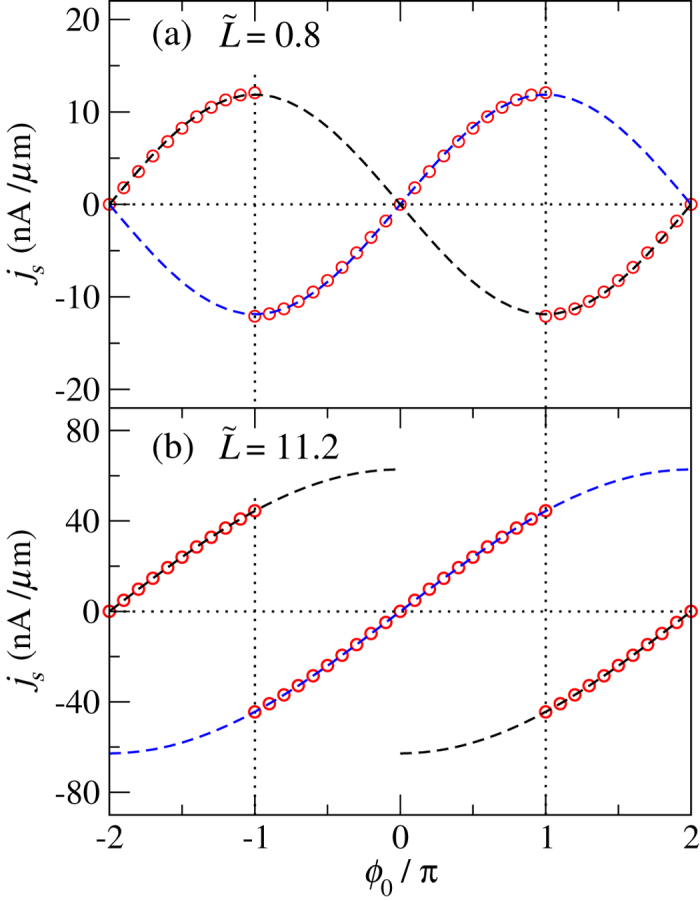
Current-phase relations (CPR) for (a) long and (b) short junctions. The red open circles show the numerically calculated supercurrent density for the lowest energy state. The blue dash lines plot 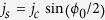 in (**a**) and 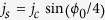 in (**b**). The black dash lines plot the complementary soltion (CS) of 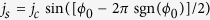 in (**a**) and 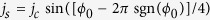 in (**b**). For *ϕ*_0_ < *π* the red circles fall on top of the blue line; the numerical calculation is consistent with the DS from our simple pictures in both the short- and the long- junction limits. For 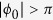, the lowest energy states turn into the CS. A jump occurs at 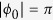 if the system can quickly relax to the lowest energy state.

The supercurrent density *j*_*s*_ can further be acquired from the phase profile by j^s^ = (*e*/*h–*) 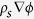; in this report, we focus on the supercurrent density at the interface. In Fig. 4 we plot the CPRs obtained from the numerical LLG calculation. The analytical result for short junction is obtained side-by-side from Eq. (4) (in dimensionless expression):


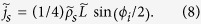


This dimensionless current density 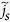 is taken with respect to eE0/h–λ. The above exhibits a critical current density of 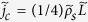; also, with *ϕ*_*i*_ = *ϕ*_0_, the expression corresponds to DS while with *ϕ*_*i*_ = *ϕ*_0_ − 2*π*sgn(*ϕ*_0_) to CS. In Fig. 4, we plot the supercurrent densities of analytic solution for both the DS (blue dash line) and the CS (black dash line), as well as the numerical calculation (red open circles). The sin(*ϕ*_0_/2) dependence in the DS is in great accordance with the numerical result (Fig. 4) for 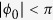 (Note that in plotting *j*_*s*_ we have intentionally set back the units to make direct comparison with the numerical results). The case of 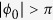 will be discussed jointly with that in the long junction.

In the long junction, the fractional solitons in Eq. (6) give rise to the supercurrent density


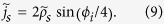


This is distinct from the current density in the short junction. The critical current is *size-independent* and is given by 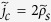. The CPR of sin(*ϕ*_*i*_/4) dependence is also qualitatively different from that of sin(*ϕ*_*i*_/2) in the short junction. The numerical and the analytical results for DS are consistent for 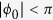, when DS is the lowest energy state. What appears to be different starts at 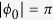, when the DS and CS become degenerate in both the short and the long junctions. As 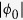 exceeds *π*, the CS replaces the DS to be the lowest energy state. The polarity of the CPR swiches. In either the long or the short junction, an abrupt commensurate-incommensurate (linear-soliton) transition as that in the Pokrovsky-Talapov model^5^,^7^,^51^,^52^ is not seen. Topological objects are present for arbitrarily small *ϕ*_0_.

**Figure 5:**
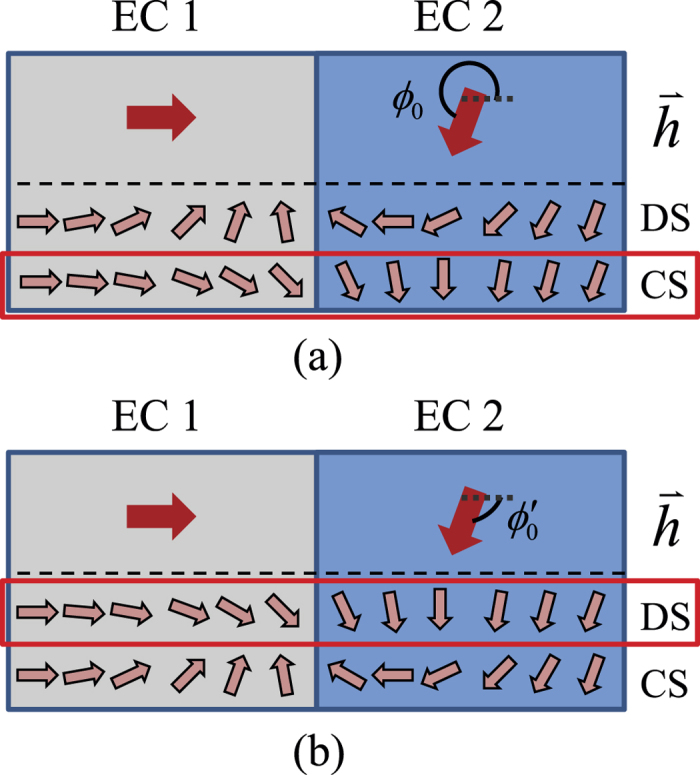
Illustration of pseudospin configurations. (**a**) The pseudospin configurations for the DS and CS with *ϕ*_0_ > *π*. The CS is the lowest energy state for *π* < *ϕ*_0_ < 2*π*. (**b**) The DS and CS switch when taking 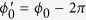. The lower energy states are circled in red in both (**a**,**b**).

The switch of the lowest energy state can best be visualized in the pseudospin picture; here a long junction is chosen for illustration in Fig. 5 but the description is general. The pseudospin Zeeman fields in EC1 and in EC2 align the local pseudospin differently and give rise to two different domains, and a wall in between (soliton). When the two domains are *not completely anti-aligned*, the local pseudospins do not wind a full circle within the wall – this corresponds to a *fractional* soliton. While the local pseudospins can be arranged into different configurations with the same *ϕ*_0_, the one with the slowest variation costs least energy. For 0 < *ϕ*_0_ < *π*, the DS (winding counter-clockwise) has the slower variation and is preferred. The DS and CS become mirror symmetric when *ϕ*_0_ equals *π*. For *ϕ*_0_ > *π*, the CS (winding clockwise) has slower variation [Fig. 5(a)] and takes over the lowest energy state. Also we recognize that the CS for any *ϕ*_0_ is genuinely the DS for another 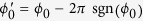 [Fig. 5(b)]. The full CPR (lowest in energy) should be the periodic replications of that between −*π* and *π*.

## Conclusion

To summarize, the excitonic Josephson junction is mapped to a pseudospin ferromagnet and described by the Landau-Lifshitz-Gilbert equation. The phase profile and current-phase relation are calculated. We find distinct behaviors in the long- and the short-junction limits. In the short junctions, the phases are essentially slanting lines with the slopes proportional to the system size. In the long junctions most interestingly, we recognize the static fractional sine-Gordon soliton – the soliton fraction (topological charge) can be tuned *continuously* by the relative phase *ϕ*_0_. In addition, there are two relevant solutions, a direct solution (DS) and a complementary solution (CS); both are present for any junction length. These two solutions are opposite in polarity and carry different energies. The DS is the lowest energy state for *ϕ*_0_ up to 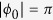. The CS then takes over as the lowest energy state for 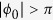. If the system can rapidly relax the excess energy, a sudden jump should appear in the current-phase relation. There is still plenty to explore in excitonic Josephson junctions: interaction of fractional solitons in multiple junctions, exotic materials as weak links and much more. This work can open up new physics in the context of excitonic superfluid.

## Additional Information

**How to cite this article**: Hsu, Y.-F. and Su, J.-J. Fractional Solitons in Excitonic Josephson Junctions. *Sci. Rep*. **5**, 15796; doi: 10.1038/srep15796 (2015).
