# Corrigendum: Fractional Solitons in Excitonic Josephson Junctions

**DOI:** 10.1038/srep46847

**Published:** 2017-06-07

**Authors:** Ya-Fen Hsu, Jung-Jung Su

Scientific Reports 5: Article number: 1579610.1038/srep15796; published online: 10 29 2015; updated: 06 24 2017

In this Article, an affiliation has been omitted for Ya-Fen Hsu. The correct affiliations are listed below:

Department of Electrophysics, National Chiao Tung University, Hsinchu 300, Taiwan.

Physics Division, National Center for Theoretical Science, Hsinchu, 30013, Taiwan.

